# FAIRification of the DMRichR pipeline: advancing epigenetic research on environmental and evolutionary model organisms

**DOI:** 10.1093/bioadv/vbaf024

**Published:** 2025-02-06

**Authors:** Wassim Salam, Marcin W Wojewodzic, Dagmar Frisch

**Affiliations:** Department of Mathematics and Computer Science, Freie Universität Berlin, 14195 Berlin, Germany; Department of Research, Cancer Registry of Norway, Norwegian Institute of Public Health, 0379 Oslo, Norway; Department of Chemical Toxicology, Norwegian Institute of Public Health, 0213 Oslo, Norway; Department of Evolutionary and Integrative Ecology, Leibniz Institute of Freshwater Ecology and Inland Fisheries, 12587 Berlin, Germany

## Abstract

**Summary:**

Bioinformatics tools often prioritize humans or human-related model organisms, overlooking the requirements of environmentally relevant species, which limits their use in ecological research. This gap is particularly challenging when implementing existing software, as inadequate documentation can delay the innovative use of environmental models for modern risk assessment of chemicals that can cause aberration in methylation patterns. The establishment of fairness in ecological and evolutionary studies is already constrained by more limited resources in these fields of study, and an additional imbalance in tool availability further hinders comprehensive ecological research.

To address these gaps, we adapted the DMRichR package, a tool for epigenetic analysis, for use with custom, non-model genomes. As an example, we here use the crustacean *Daphnia*, a keystone grazer in aquatic ecosystems. This adaptation involved the modification of specific code, computing three new species-specific packages (BSgenome, TxDb, and org.db), and computing a CpG islands track using the makeCGI package. Additional adjustments to the DMRichR package were also necessary to ensure proper functionality. The developed workflow can now be applied not only to different *Daphnia* species that were previously unsupported but also to any other species for which an annotated reference genome is available.

**Availability and implementation:**

Code and data are available at https://github.com/wassimsalam01/DMRichR-FAIRification and at https://github.com/folkehelseinstituttet/DMRichR-FAIRification as well as DOI 10.5281/zenodo.13366959. This work is open-source software available under the GNU Affero General Public License (AGPL) version 3.0.

## 1 Introduction

Epigenetic research is a crucial field in ecological research for understanding how organisms adapt to environmental challenges (Thiebaut *et al.* 2019, McGuigan *et al.* 2021, Lamka *et al.* 2022) as well as for application in ecotoxicological studies that involve non-model species (Vandegehuchte and Janssen 2014, Šrut 2021). However, the related bioinformatics tools are predominantly oriented toward humans or model organisms for humans, often neglecting the requirements for environmental model organisms. This disparity hinders their development and application in ecological research or modern risk assessment for chemicals. Additionally, a lack of clear documentation or the absence of necessary dependencies further complicates the implementation of existing software for these organisms.

Differential genome-wide methylation analysis involves the comparison of methylation patterns across different conditions to understand the impacts on gene regulation and consequently gene expression (Parle-Mcdermott and Harrison 2011, Li and Tollefsbol 2021). Currently, a multitude of tools exists that are suitable for differentially methylated region (DMR) analysis, each of which makes use of a different differential methylation test. These include but are not limited to: Fisher’s exact test, BSmooth, MethylKit, MethylSig, DSS, Metilene, RADMeth, and Biseq. Each tool uses a unique approach, with none of them consistently outperforming others during benchmark testing (Piao *et al.* 2021).

One recent tool that stands out for its robust use of a Bayesian framework is DMRichR (v1.7.8) (Hansen *et al.* 2012, Korthauer *et al.* 2019, Laufer *et al.* 2021). It is a powerful resource in epigenetic research which contrasts with other DMR analysis tools by combining both the dmrseq (v1.15.1) and bsseq (v1.38.0) algorithms to identify DMRs with greater precision and accuracy. It improves analysis by incorporating prior knowledge and probabilistic models to better capture true methylation changes, thus reducing false positives. This method prioritizes methylation sites with higher coverage, using them to infer nearby sites with less data. To identify DMRs, this approach focuses on the comparison of groups of samples (e.g. treatments) rather than within-group levels and allows the analysis of samples even at a lower sequencing depth of 1-5× (Laufer 2023). DMRs are identified in two steps: first, pooling and weighting data from high-coverage sites to detect differences, and then statistically testing these regions to identify significant changes across the genome (Korthauer *et al.* 2019, Laufer 2023). The biggest notable drawback of DMRichR is that it is primarily available for typical model species and its application can be challenging for researchers with limited bioinformatics experience. As of its last update in October 2023, only 15 species are supported, which include *Homo sapiens* and *Mus musculus*, while excluding many environmentally important species or evolutionary models such as *Daphnia*.

One of such important additions to the suite of taxa to be used with DMRichR is aquatic organisms. *Daphnia* are keystone organisms in aquatic ecosystems, serving as a vital link in the food web between primary producers and higher trophic levels (Miner *et al.* 2012, Ogorelec *et al.* 2021). In the last decade, the planktonic crustacean *Daphnia* has emerged as an important model in ecological and evolutionary research and is supported by institutions such as the National Institutes of Health (NIH) (Ebert 2005, 2022). Despite their extensive use in genetic, ecotoxicological, and ecological research (Colbourne *et al.* 2022), genomic resources are typically built for *Daphnia pulex* and *Daphnia magna*, but even for these, bioinformatic tools remain scarce. Although *Daphnia* has been highlighted as a promising epigenetic model (Harris *et al.* 2012, Wojewodzic and Beaton 2017, Agrelius and Dudycha 2025), more empirical studies are needed to address the eco-evolutionary significance of methylation. Providing the implementation of DMRichR for these taxa will facilitate access to the pipelines in the research community, and could potentially also drive modern chemical risk assessment when epigenetic effects are of interest.

**Figure 1.**
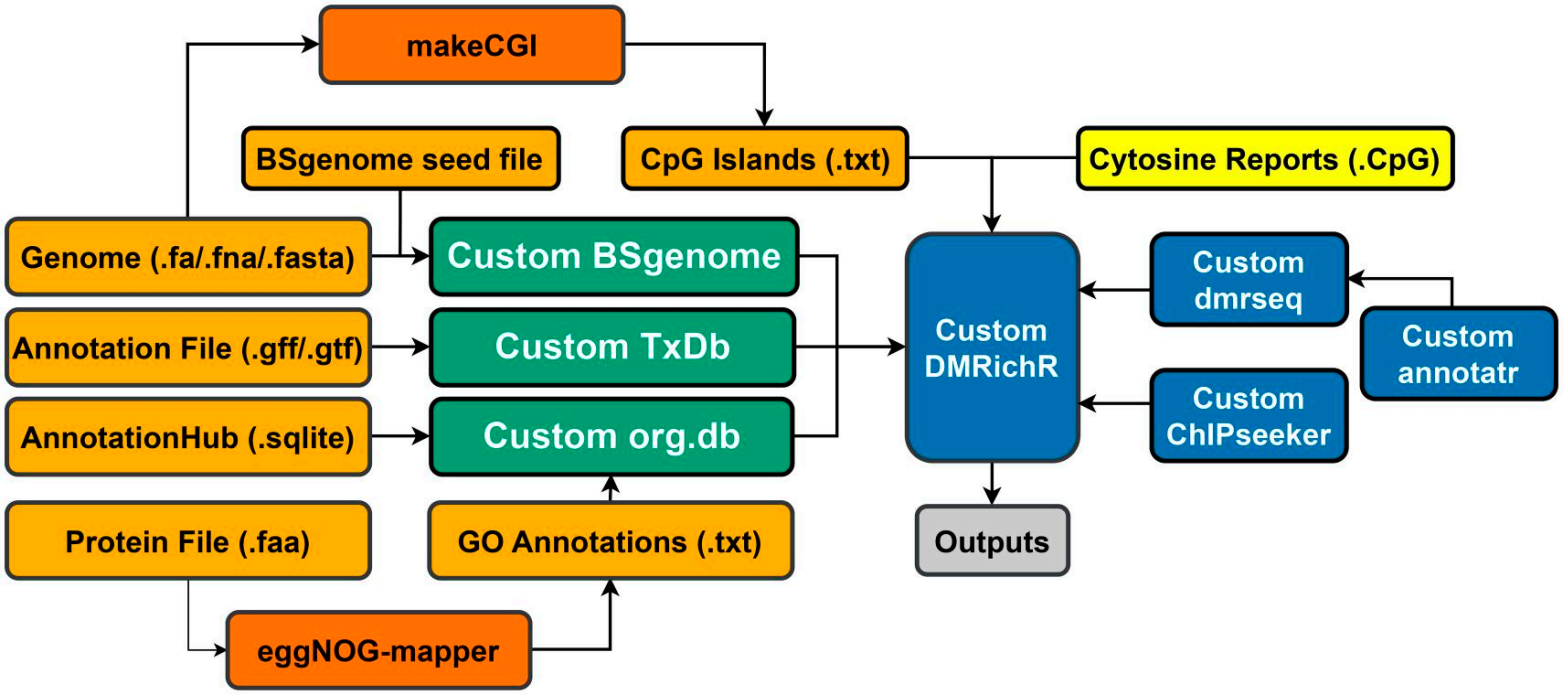
Steps required to adapt DMRichR to a new organism with an annotated genome. *Yellow*: User input files (methylation calls (.CpG)) resulting from a Whole-Genome Bisulfite Sequencing (WGBS) experiment; *Light-orange:* User input files with information on the reference genome (BSgenome seed files, CpG islands, .fa/.fna/.fasta, .gff/.gtf, .sqlite, .faa, GO Annotations); *Dark-orange*: Intermediary tools for producing input files (makeCGI, eggNOGmapper); *Green*: Newly-computed species-specific packages (Custom BSgenome, Custom TxDb, Custom org.db); *Blue*: Modified versions of DMRichR and additional packages used within it (Custom DMRichR, Custom dmrseq, Custom ChIPseeker, Custom annotatr); *Grey*: Outputs generated by DMRichR, which include Blocks, Differentially Methylated Regions (DMRs), Smoothed Individual Methylation Values, Heatmaps.

The use of custom genomes with the R package DMRichR can be achieved by making appropriate modifications in the code of annotationDatabases.R (Laufer 2023). This requires in a first step the computation of three new packages (Fig. 1): BSgenome, TxDb, and org.db (for description of functionality see below). Next, a CpG islands track must be computed, which can be done using the makeCGI package (Irizarry *et al.* 2009; Wu *et al.* 2010). Lastly, a few modifications have to be made to packages used within DMRichR’s code, namely within Dmrseq, Annotatr, and ChIPseeker.

## 2 Implementation

An overview of the workflow is provided in Fig. 1. While here we executed the workflow specifically for *D.pulex*, it could also be applied more generally for any other species with an annotated reference genome following the same steps. The R code (v4.3.2) (R Core Team 2023) associated with each package’s computation can be found in Table 1, link 1. In the next sections, we describe the generation or modification made to provide all packages and steps for DMRichR to function with the *D.pulex* genome and the cytosine report files produced by Bismark (Krueger and Andrews 2011).

**Table 1.** Links to online sources.

Link	Item	Github page
1	R code for computing packages needed for DMRichR	https://github.com/wassimsalam01/DMRichR-FAIRification/blob/main/r-scripts/computeDatabases.R
2	BSgenome seed file	https://github.com/wassimsalam01/DMRichR-FAIRification/blob/main/r-scripts/BSgenome.Dpulex.NCBI.ASM2113471v1-seed
3	*D. pulex* BSgenome package	https://github.com/wassimsalam01/DMRichR-FAIRification/blob/main/case-study/BSgenome.Dpulex.NCBI.ASM2113471v1_1.0.0.tar.gz
4	*D. pulex* TxDb package	https://github.com/wassimsalam01/DMRichR-FAIRification/blob/main/case-study/TxDb.Dpulex.NCBI.ASM2113471v1.knownGene_1.0.tar.gz
5	*D. pulex* org.db package	https://github.com/wassimsalam01/DMRichR-FAIRification/blob/main/case-study/org.Dpulex.eg.db_1.0.tar.gz
6	Hao Wu Lab	https://www.haowulab.org/software/makeCGI/index.html
7	makeCGI execution	https://github.com/wassimsalam01/DMRichR-FAIRification/blob/main/r-scripts/makeCGI.R
8	*D. pulex* CpG Islands	https://github.com/wassimsalam01/DMRichR-FAIRification/blob/main/case-study/CGI-Dpulex.txt
9	Custom DMRichR change log	https://github.com/wassimsalam01/DMRichR/commits/master/R
10	Custom dmrseq change log	https://github.com/wassimsalam01/dmrseq/commits/master/R
11	Custom annotatr change log	https://github.com/wassimsalam01/annotatr/commits/devel/R
12	Custom ChIPseeker change log	https://github.com/wassimsalam01/ChIPseeker/commits/devel/R
13	Custom DMRichR case study test run	https://github.com/wassimsalam01/DMRichR-FAIRification/blob/main/case-study/DMRichR-Testing.R
14	Cytosine reports (.CpG_report.txt.gz)	https://github.com/wassimsalam01/DMRichR-FAIRification/tree/main/case-study

### 2.1 BSgenome

The BSgenome package enables users to efficiently manage and analyze whole genome sequences. It provides tools for tasks such as extracting genomic sequences and conducting genome-wide analyses. Apart from DNA methylation analysis, this has additional value for use in other applications such as sequence alignment, variant calling, histone modification (ChiPseq) analysis, and motif discovery (Pagès 2024). The first step is to write a “seed file” following BSgenome guidelines (Pagès 2024), which contains package metadata and instructions on how the package should be compiled. The *D.pulex* ASM2113471v1 genome assembly (NCBI 2021) was used to produce the seed file BSgenome.Dpulex.NCBI.ASM2113471v1-seed (Table 1, link 2). Following recommended naming conventions, the package BSgenome.Dpulex.NCBI.ASM2113471v1 was computed (Table 1, link 3).

### 2.2 TxDb

Using either a GFF (General Feature Format) or GTF (Gene Transfer Format) file, a transcript annotation package can be computed. It contains transcript-related annotations (like exons, introns, UTRs), which in addition to being a central package for DMRichR, may be useful for applications such as transcriptomics, RNA-Seq data analysis, Chip-Seq annotation, and gene structure analysis. The *D.pulex* GTF file (NCBI 2022) was used, and in a two-step process, the package TxDb.Dpulex.NCBI.ASM2113471v1.knownGene was computed (Table 1, link 4).

### 2.3 org.db

This package contains mappings between a central identifier (e.g. Entrez gene IDs) and other identifiers (e.g. gene symbol, gene name, gene ontology, chromosome) (https://bioconductor.org/help/course-materials/2014/useR2014/Integration.html). The AnnotationHub package already contains a *D.pulex* database in SQLite format, with “GID” (Entrez ID) as a central key for its tables. A GO (Gene Ontology) annotation file was obtained by passing the *D.pulex* protein file (protein.faa.gz) (NCBI 2022) to eggNOG-mapper (Cantalapiedra *et al.* 2021) with default options. By combining these components we computed org. Dpulex.eg.db, following org.db package naming conventions (Table 1, link 5).

### 2.4 CpG islands

By using the makeCGI package (Irizarry *et al.* 2009; Wu *et al.* 2010), CpG Islands of any available annotated genome can be de novo discovered to build a CpG islands track, which is an integral component for the functionality of the DMRichR analysis. Computation of the BSgenome package is a prerequisite to this step (Fig. 1), as makeCGI loads the specified genome from the latter. The posterior probability is one of many parameters that the user can modify, which affects how the package decides what defines a CpG Island. From the CGI files of different organisms already available on the Hao Wu Lab website (Table 1, link 6), a posterior probability of 0.99 was chosen for all genomes except for that of the fruit fly *Drosophila melanogaster* (0.975). makeCGI was executed with BSgenome.Dpulex.NCBI.ASM2113471v1 (Table 1, link 7), with a chosen posterior probability similar to that of *D.melanogaster*, because the *Daphnia* genome has a higher resemblance to that of the fruit fly than to the other listed genomes. The genome of *Daphnia*, like that of the fruit fly, is characterized by a small number of methylated bases (Asselman *et al.* 2016, Kusari *et al.* 2017). A text file containing CpG Islands entries was therefore produced (Table 1, link 8). These parameters might need further tuning for other species.

### 2.5 Modifications of DMRichR and additional packages

Multiple snippets of code were adjusted within the DMRichR package. Below we provide a short overview of the changes, which can be viewed in detailed in Table 1, link 9:

- Added new genome “Dpulex” and integrated CGI annotations.
- Integrated new BSgenome, TxDb, and org.db packages in “annotationDatabases.R.”
- Modifications were made to arguments such as “minInSpan,” “bpSpan,” “maxGapSmooth,” “maxGap,” “minNumRegion,” and “blockSize.” This was done because *D.pulex* has not only a significantly smaller size but also low and sparse methylation. Lowering the threshold of default arguments allows for more methylation data to be captured.
- Added argument “cytosineReportFormat” (default NULL). Setting a value of “nf-core/methylseq” (Ewels *et al.* 2023) would enable DMRichR to process cytosine reports generated by nf-core/methylseq which are produced with a slightly different naming convention than when produced by Bismark.
- The packages dmrseq, annotatr, and ChIPseeker were adjusted to allow the integration of the *D.pulex* genome. The respective changes can be seen in Table 1, links 10, 11, and 12.

All analyses were performed using R Statistical Software (v4.3.2) (R Core Team 2023) and Bioconductor (v3.18) (Huber *et al.* 2015). The BSgenome seed-file for *D.pulex*, the code used to compute the above-mentioned packages and the CpG islands list, as well as the sample data (cytosine reports) used in the DMRichR test-run are publicly available. For ease of use, the installations of the computed *D.pulex* packages (BSgenome, TxDb, and org.db) were seamlessly integrated into the custom DMRichR package. However, they are as well readily available to use independently of DMRichR. This can prove useful for specific applications, some of which are mentioned in the respective package sections.

## 3 Case study

**Figure 2.**
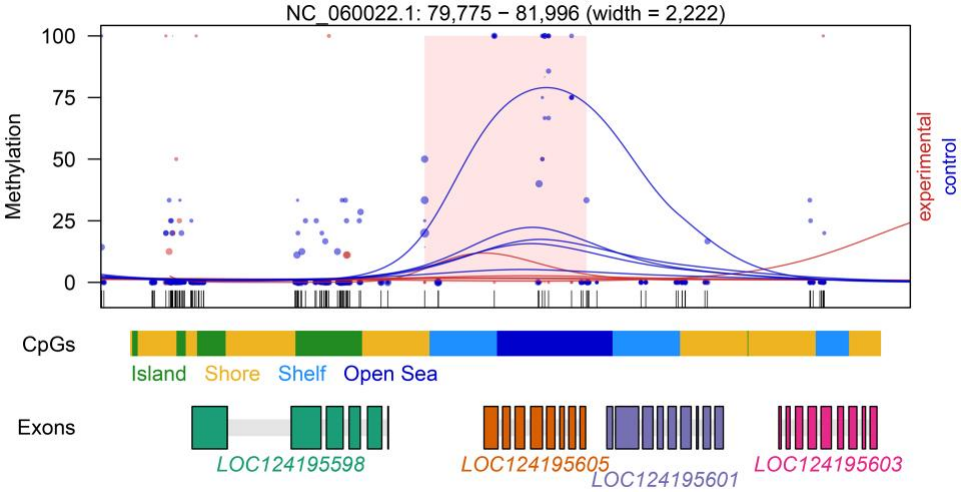
DMR plot displaying a DMR consisting of 13 CpGs with 23% hypomethylation in experimental samples compared to control samples. The methylation level of each CpG site in an individual sample is shown as a point, with its size directly proportional to its coverage. Smoothed methylation levels are represented by lines, separately for control and experimental samples. A track of CpG and gene annotations are additionally displayed under the plot, retrieved from the computed CGI track and org.db package, respectively.

Proper functionality of the modified R package DMRichR is demonstrated using sample data from an unpublished study involving Whole-Genome Bisulfite Sequencing data from *Daphnia pulicaria*, a member of the *D.pulex* species complex (Dudycha and Tessier 1999). The provided input example presents a cytosine report generated from 10 samples (5 control versus 5 experimental), and for demonstration purposes has been reduced to report methylation only on chromosome NC_060022.1. The code for this test run is provided in Section 4, which contains instructions on setup and installation. Detailed instructions about how to test the customized DMRichR package can be found in Table 1, link 13. The cytosine reports of each sample (Table 1, link 14), which are used as input for DMRichR, were produced by nf-core/methylseq (v2.4.0) (Ewels *et al.* 2023). The DMR plot shown in Fig. 2 below displays one of many DMRs obtained by this case study run, which successfully demonstrates the FAIRification of the DMRichR pipeline.

## 4 Conclusion

Integrating support for the *D.pulex* genome into the DMRichR package represents a significant advancement in the field of ecological and evolutionary genomics. It strengthens the capacity for high-resolution analysis of DNA methylation patterns in *D.pulex*, and enhances the possibility of using whole genome methylation in a modern risk assessment for chemicals. The incorporation of support for the *D.pulex* genome to DMRichR thus allows researchers to leverage this tool’s robust functionalities to investigate epigenetic modifications and efficiently use sparse information across different treatments with greater precision. This adaptation not only facilitates deeper insights into the adaptive mechanisms and environmental responses of *D.pulex* but also creates possible use for risk assessment using epigenetics that is still underexplored in ecological studies.

The workflow described here sets a precedent for similar enhancements in other species. The process involves the careful annotation of the target species’ genome, followed by integration into the DMRichR framework, thereby enabling the broader scientific community to extend these powerful analytical capabilities to a diverse array of organisms. With an annotated reference genome, increasingly available for many non-model species, the workflow we have described and tested here is particularly beneficial for researchers studying methylation patterns in ecologically and evolutionary significant species, as it bridges the gap between advanced bioinformatics tools and ecological research, fostering a more comprehensive understanding of epigenetic regulation in varied environmental contexts. Lastly, the packages produced in this work contribute not only to the advancement of differential methylation analysis but also to other applications improving FAIRness of these tools for environmental research.

## Data Availability

Code and data are available at https://github.com/wassimsalam01/DMRichR-FAIRification and at https://github.com/folkehelseinstituttet/DMRichR-FAIRification, as well as *at DOI 10.5281/zenodo.13366959*. This work is open-source software available under the GNU Affero General Public License (AGPL) version 3.0.
